# Translation, cross-cultural adaptation, and validation of the Palliative Care Knowledge Questionnaire for PEACE (PEACE-Q) in Brazilian Portuguese

**DOI:** 10.31744/einstein_journal/2025AO1243

**Published:** 2025-07-30

**Authors:** João Paulo Aureliano Silva, Aleida Nazareth Soares, Adriana Silvina Pagano, Cristiana Guimarães Paes Savoi, Alexandre Ernesto Silva, Alexandre Sampaio Moura

**Affiliations:** 1 Graduate Program in Medicine and Biomedicine Faculdade de Saúde Santa Casa BH Belo Horizonte MG Brazil Graduate Program in Medicine and Biomedicine, Faculdade de Saúde Santa Casa BH, Belo Horizonte, MG, Brazil.; 2 Universidade Federal de Minas Gerais Graduate Program in Linguistics and Applied Linguistics Belo Horizonte MG Brazil Graduate Program in Linguistics and Applied Linguistics, Universidade Federal de Minas Gerais, Belo Horizonte, MG, Brazil.; 3 Hospital da Baleia Department of Palliative Care Belo Horizonte MG Brazil Department of Palliative Care, Hospital da Baleia, Belo Horizonte, MG, Brazil.; 4 Universidade Federal de Minas Gerais Postgraduate Program in Nursing Divinópolis MG Brazil Postgraduate Program in Nursing, Universidade Federal de Minas Gerais, Divinópolis, MG, Brazil.

**Keywords:** Palliative care, Internship and residency, Education, medical, Clinical competence, Validation study as topic, Psychometrics, Surveys and questionnaires

## Abstract

**Objective:**

To translate, cross-culturally adapt, and validate the Palliative Care Knowledge Questionnaire for PEACE (PEACE-Q) in Brazilian Portuguese.

**Methods:**

This study followed five steps: translation, back-translation, cultural adaptation, pre-test, and test-retest. Pre-test (n=20) and test-retest (n=63) were conducted on medical residents from Santa Casa Hospital in Belo Horizonte, Brazil. We determined the content validity index, Kuder-Richardson-20 (KR-20) value, and intraclass correlation coefficient. The performances of residents with different years of training and specialty areas were compared using the Kruskal-Wallis and Mann-Whitney tests.

**Results:**

The translated version of the instrument comprised 33 items divided into nine domains, with a content validity index of 0.95. The test-retest on medical residents showed an internal consistency (KR-20) of 0.60 (95% confidence interval (95%CI)=0.54-0.66) and intraclass correlation coefficient of 0.71 (95%CI=0.51-0.82). Medical residents of clinical specialties showed better overall scores than those of surgical or mixed specialties (median scores [IQR]: 27.0 [25.0-28.0], 26.0 [23.0-28.0], and 23.5 [21.5-25.5], respectively; [p=0.013]). Regarding year of training, medical residents on year three or beyond had higher scores in the "Opioid Side Effects" domain than those on the first 2 years [median scores (IQR): 2.0 (2.0-3.0) *versus* 2.0 (1.0-2.0); p=0.03].

**Conclusion:**

The Brazilian version of the PEACE-Q demonstrated acceptable internal consistency and moderate reliability and appears appropriate for assessing the palliative care knowledge of Brazilian medical residents.

## In Brief

The PEACE-Q was translated into Brazilian Portuguese and validated for use with medical residents. The Brazilian version of the instrument showed acceptable consistency, moderate reliability, and appropriate validity for assessing palliative care knowledge among this population.

## Highlights

■ Performance was < 70% in 10 of the 33 items.
■ Lowest performance domains were Oncologic Pain and Opioid Side Effects.
■ Residents in clinical specialties outperformed those in surgical or mixed specialties.
■ Residents with ≥3 years of training outperformed those on their first 2 years.

## Graphical abstract

**Figure:**
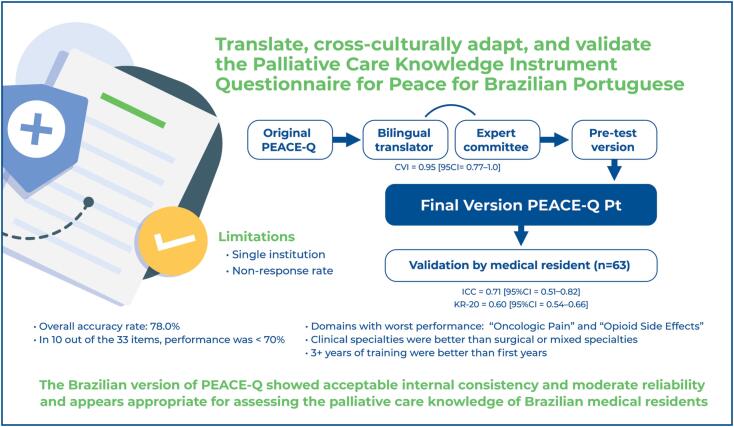

## INTRODUCTION

Palliative care is a specialized form of care associated with life-threatening illnesses that aims to prevent and relieve health-related suffering of adults, caregivers, and their families. It should be provided by a multidisciplinary team, offering a comprehensive and person-centered approach to address physical, psychological, social, and spiritual suffering.^(1-4)^

The primary conditions associated with the need for palliative care include cancer, cerebrovascular disease, and dementia. When properly implemented, palliative care can reduce the unnecessary use of health resources, lower direct hospitalization costs, and improve patients’ quality of life.^(5-7)^

A significant barrier to providing palliative care is the lack of preparedness among physicians.^(8)^ For example, healthcare professionals, including physicians and nurses, often have misconceptions regarding the use of pain management medications.^(9)^ Palliative care training programs have increased worldwide; however, their content remains significantly heterogeneous. Moreover, most undergraduate medical curricula lack palliative care education.^(4,8,10,11)^ Therefore, assessing the palliative care knowledge of medical residents using validated instruments can help identify gaps and improve the design of educational programs.

Many instruments have been developed to assess the knowledge or self-efficacy of healthcare professionals in end-of-life care, with the goal of understanding the quality of palliative care provided to patients.^(12)^A systematic review analyzed the psychometric properties of such instruments and listed the Palliative Care Knowledge Test (PCKT) and the Palliative Care Knowledge Questionnaire for PEACE (PEACE-Q) as the preferred choices.^(12)^ Both instruments were developed in Japan; however, the psychometric properties of PCKT were assessed only among nurses.^(13)^

The PEACE-Q was developed to assess the palliative care knowledge of physicians participating in the PEACE ("*Palliative Care Emphasis Program on Symptom Management and Assessment for Continuous Medical Education*") initiative, which aimed to improve palliative care in Japan.^(14)^ The PEACE-Q showed high-quality evidence for all the psychometric properties except for content validity, which was classified as ‘moderate.’^(12)^

The PEACE-Q has been used in different contexts and as the basis for a new assessment instrument developed in Germany;^(15-17)^ however, it has not yet been translated into Brazilian Portuguese.

## OBJECTIVE

This study aimed to translate, cross-culturally adapt, and validate the PEACE-Q in Brazilian Portuguese.

## METHODS

### Study design

This study followed the five steps proposed by Ramada-Rodilla et al.:^(18)^ forward translation of the PEACE-Q into Portuguese, back translation into English, cultural adaptation, pre-test, and test-retest. Information on the sociodemographic and training characteristics of medical residents was obtained using an online questionnaire. This study was conducted at Santa Casa BH, Brazil, between February and May 2023, and all participants provided informed consent. This study was approved by the Institutional Review Board of *Santa Casa* BH (CAAE: 63574522.6.0000.5138; # 5.694.876). Consent for translation and adaptation was obtained from the author of the original version of the instrument.

### Participants

A convenience sample of medical residents enrolled in postgraduate training at Santa Casa BH was recruited during the study period. Medical residents were excluded if they did not respond to the re-test within 14 days. According to the Consensus-based Standards for the Selection of Health Measurement Instruments (COSMIN), the required sample size for the validation of measurement instruments in methodological studies is 50 individuals.^(19)^

### Procedures

#### Translation and cultural adaptation

Two translators (T1 and T2) initially translated the questionnaire into Portuguese and created a synthesized version (T1-T2). Five items (2, 3, 31, 32, and 33) from the original instrument were identified as specific to the Japanese healthcare context and were replaced. The authors developed new replacement items while maintaining a meaning close to the original context.

The replacement questions were included in the T1-T2 version of the instrument and back-translated (BT1 and BT2) by two bilingual translators (BT1 and BT2). After completing this step, the two back-translated versions were compared with the original English version of the questionnaire. Upon confirming the absence of relevant disagreements, a final Portuguese version (Supplementary Material - PEACE-Q Br) was obtained and used in the subsequent steps.

A committee of nine experts in palliative care received the original version of the PEACE-Q and the final translated version that contained five items adapted to the Brazilian context. The experts assessed each item of the translated instrument for conceptual, idiomatic, semantic, and experiential equivalence to the original version, providing a score as follows: (i) one star indicated a need for complete retranslation; (ii) two stars, need for partial retranslation with significant editing; (iii) three stars, need for partial retranslation with optional editing to improve style; and (iv) four stars, no need for any editing or retranslation. Committee members were also asked to comment on the translated items and provide suggestions for improving the instrument.

#### Pre-test

The pre-test was conducted on medical residents who did not participate in the test-retest steps. Participants were instructed to read all the items and report their impressions of clarity and ambiguity.

#### Test and retest

The study participants answered the PEACE-Q Br to assess reliability and construct validity. Seven days after the initial response, the same questionnaire was re-administered to the participants.

### Data analysis

Internal content validity was evaluated using the content validity index (CVI), and reliability was assessed using the intraclass correlation coefficient (ICC).^(20)^ For instruments previously constructed and validated in other cultures, a minimum CVI of 0.78 was considered acceptable. Additionally, a recommended ICC value >0.70 indicated good test-retest reliability.^(21)^

Internal consistency analysis was performed using the Kuder-Richardson Formula 20 (KR-20). Non-parametric Kruskal-Wallis and Mann-Whitney U tests were used to compare the overall and domain-specific scores among the different groups.

The classification of residency programs into clinical, surgical, or mixed was based on the frequency of the residents’ involvement in the operating room (OR): (i) clinical (no direct contact with the OR), (ii) surgical (primarily working in the OR), and (iii) mixed (specialties that require more hours in clinics and outpatient settings but also involve working in the surgical center).

## RESULTS

### Translation and cultural adaptation

The expert committee consisted of nine palliative care specialists, whose characteristics are presented in table 1. After the committee's first round of analysis, a minimum CVI threshold of 80% was achieved (CVI: 0.95 [0.77-1.0]). The suggestions provided were analyzed, and minor changes were made. The pre-test was conducted on 20 medical residents. No item received clarification requests from more than 15% of the participants.

**Table 1:** Characteristics of the expert committee participants

Variables		Participants’ characteristics
Female, n (%)		7 (77.8)
Mean age (SD), years		39.8 (5.9)
Highest level of education, n (%)		
	Residency/specialization	6 (66.7)
	Master's degree	3 (33.3)
Specialization or field of practice, n (%)		
	Palliative care only	5 (55.6)
	Palliative care + other medical specialty	4 (44.4)
Mean time of medical experience (SD), years		15.4 (6.3)
Mean time of palliative care experience (SD), years		8.7 (5.1)
English proficiency, n (%)		
	Intermediate	6 (66.7)
	Advanced	3 (33.3)

SD: standard deviation.

### Internal consistency and test-retest reliability

A total of 102 medical residents completed the questionnaire; however, 39 were excluded from the study because they did not respond to the retest, resulting in a final sample of 63 participants.

The participants were predominantly women (61.9%), with a mean age (±SD) of 27.6 (± 3) years. Most participants (85.7%) reported having never participated in a specific palliative care course. Table 2 shows the distribution of medical residents according to specialty.

**Table 2:** Characteristics of study participants

Variables		Participants’ characteristics
Sex, n (%)		
	Male	24 (38.1)
	Female	39 (61.9)
Mean age (SD), years		27.6 (3.0)
Previous contact with palliative care, n (%)		
	Yes, during medical graduation	6 (9.5)
	Yes, during medical residency/fellowship	3 (4.8)
	No, up to the present moment	54 (85.7)
Feel confident in prescribing morphine, n (%)		
	Yes	43 (68.3)
	No	20 (31.7)
Medical Residency Program, n (%)		
	Internal Medicine	10 (15.8)
	General Surgery	6 (9.5)
	Endocrinology	6 (9.5)
	Intensive Care Medicine	6 (9.5)
	Orthopedics	5 (7.9)
	Others	30 (47.3)

SD: standard deviation.

The ICC was 0.71 (95%CI= [0.51-0.82]), and the instrument's internal consistency (KR-20) was 0.60 (95%CI= [0.54-0.66]).

### Preliminary profile of medical residents’ knowledge of palliative care

The mean overall accuracy rate of medical residents on the PEACE-Q Br was 78.0% (SD=20.2) and 77 % (SD=21) in the test and retest, respectively. In 10 of the 33 items in the PEACE-Q questionnaire, the performance of medical residents was <70%. Most of these items were related to the "Oncologic Pain" and "Opioid Side Effects" domains (Table 3).

**Table 3:** Mean scores of medical residents’ performance for each item and domain on the PEACE-Q Br

Questions		Correct answer	Percentage of accuracy	
			Test %	Retest %
Domain 1: Philosophy of palliative care			82	84
	1. Palliative Care is a synonym for end-of-life care	F	89	92
	2. Among Brazilians, the primary feelings associated with cancer are fear of death and pain	T	97	95
	3. Opioid consumption for pain in Brazil is lower than in Mexico, Argentina, and Chile	T	60	63
Domain 2: Oncological pain			62	60
	4. When oncological pain is intense, one of the medications from the third step of the WHO analgesic ladder is used as an initial analgesic	T	63	57
	5. When prescribing opioids begins, all non-opioid analgesics should be discontinued	F	98	97
	6. Morphine is safely used in patients with renal insufficiency	F	41	49
	7. The opioid rescue dose is 5% of the total daily dose	F	62	51
	8. Since there is no tolerance for opioid-induced nausea, an antiemetic should be prescribed to all patients	F	84	83
	9. The total daily opioid dose is increased by 10% if pain is not relieved	F	33	27
	10. Opioid rotation or substitution should be considered when there is difficulty in increasing the dose due to adverse effects	T	89	90
	11. About 10% of patients with controlled basal pain experience breakthrough pain	F	17	10
	12. Invasive dental procedures should be avoided during bisphosphonate treatment	T	67	75
Domain 3: Opioid side effects			65	64
	13. Nausea and vomiting induced by opioids occur in 80% or more of patients who use them	F	62	56
	14. It is necessary to associate a laxative with oral opioids because most patients using them experience constipation	T	57	67
	15. Opioids cause dependence in 0.2% or less of cancer patients under careful monitoring	T	75	70
Domain 4: Dyspnea			80	73
	16. If a patient has dyspnea, their PaO_2_ is less than 60 mmHg	F	84	81
	17. Morphine is effective for dyspnea	T	76	73
	18. If the ambient temperature is kept higher (warm), a patient with dyspnea often feels relief	F	79	65
Domain 5: Nausea and vomiting			80	81
	19. The neurotransmitters in the vomiting center are dopamine, histamine, acetylcholine, and serotonin	T	84	84
	20. When the leading cause of nausea is hypercalcemia, the administration of bisphosphonates is a valuable treatment for relieving this symptom	T	62	68
	21. Metoclopramide can cause akathisia	T	95	92
Domain 6: Psychological distress			95	95
	22. When a patient has a high level of psychological distress, it is recommended that doctors assess whether the patient has suicidal intentions	T	100	97
	23. When the patient has suicidal intentions, psychiatric evaluation is recommended	T	98	97
	24. Anxiolytics are among the valuable medications for patients experiencing psychological distress	T	87	90
Domain 7: Delirium			86	85
	25. Delirium occurs owing to medications or organic causes	T	89	84
	26. Benzodiazepines should be the first choice for treating delirium	F	90	89
	27. It is better to keep the room of a patient with delirium very dark so that he/she can sleep well	F	78	83
Domain 8: Communication			91	92
	28. An open question cannot be answered with a simple "yes" or "no" and requires a free response based on the patient's feelings	T	89	95
	29. When doctors communicate bad news, they should inquire about the patient's concerns and understanding of the illness	T	98	97
	30. It is better to use the word ‘cancer’ repeatedly when informing the patient about their malignant disease	F	86	83
Domain 9: Palliative care in the community			95	95
	31. According to the Ministry of Health, palliative care, including primary care, should be provided at any point in the SUS health care network	T	97	95
	32. SUS supplies opioids to patients in palliative care who require this class of medications	T	95	97
	33. According to the Ministry of Health, patients in palliative care with cancer should have 24-h urgent/emergency medical assistance available in the institution where they are enrolled	T	94	92

PEACE-Q Br: Portuguese version of the Palliative Care Knowledge Questionnaire for PEACE; PaO_2:_ partial pressure of oxygen in the arterial blood; WHO: World Health Organization; SUS: Brazilian Unified Health System.

The overall performance of residents in clinical specialties was higher than that of those in surgical or mixed specialties (median scores [IQR]: 27.0 [25.0-28.0], 26.0 [23.0-28.0], and 23.5 [21.5-25.5], respectively; [p=0.013]).

Regarding the year of training, residents with ≥3 years of training (n=22) performed better than those in the first 2 years of training (n=41) in the domain "Opioid Side Effects" (2.0 [2.0-3.0] *versus* 2.0 [1.0-2.0]; p=0.03). No statistically significant difference was observed between these groups in the overall score (25.0 [13.0-28.0] *versus* 23.0 [17.0-27.0]; p=0.219).

Regarding previous experience with palliative care, 14 (22.2%) residents who reported caring for ≥10 patients in palliative care in the last 6 months performed better overall than the 30 residents who cared for ≤10 patients (median [IQR]: 27 [26-29] *versus* 26 [23-27]; p=0.013). Additionally, 43 (68.3%) participants who reported being confident in prescribing morphine performed better in the "Side Effects of Opioids" domain compared to those who felt insecure (median [IQR]: 2.0 [2.0-3.0] *versus* 2.0 [1.0-2.0]; p=0.044). No difference was observed between these two groups in the "Oncological Pain" domain (p=0.825).

## DISCUSSION

Our study results showed that the Portuguese version of the PEACE-Q (PEACE-Q Br) was reliable and valid for investigating medical knowledge regarding palliative care among resident physicians.

The stability of the translated instrument, as estimated by the ICC, was slightly lower than that obtained in the validation study of the original version of the PEACE-Q (0.84).^(14)^ However, the ICC of the PEACE-Q Br was above 0.7, which is considered the minimum satisfactory value.^(22)^ Similarly, the internal consistency of the PEACE-Q Br was lower than that obtained in the validation study of the original instrument; however, the obtained value was considered satisfactory.^(22)^

The overall accuracy rate of the medical residents in our study was slightly higher than that found in a study that assessed the original version of the PEACE-Q (mean accuracy rate of 78.0% [SD=20.2] for the test, and 77% [SD=21] for the retest).^(14)^ Performance in the "Community" domain in our study was substantially higher than that observed in the validation of the original version of the instrument (where 60% of participants had correct responses).^(14)^Notably, the questions in this domain had to be reformulated to reflect the Brazilian healthcare context and might have been less challenging than the original items.

The domains with the highest performance (95% correct responses) were "palliative care in the community" and "psychological distress." The good performance observed in the "psychological distress" domain in our study was similar to that of the original study (83.6%).^(14)^

Among the items related to symptom management, the largest knowledge gap was observed in pain management. This lack of knowledge may be explained by the limited content on this topic in medical education and the stigma associated with opioids owing to the risk of dependence.^(9,23)^ The low performance in these domains is concerning because opioids are essential in palliative care for the effective reduction of pain and dyspnea.^(24,25)^

Studies have shown that approximately one-third of students report not receiving sufficient information on how to deal with pain, highlighting the need for improved teaching of pain management in medical schools. The limited experience with opioid prescriptions can be attributed to the relatively low opioid consumption in Brazil compared to that in developed countries.^(9,23)^

The knowledge demonstrated by residents on their first 2 years of training reflects the quality of education offered in medical schools, showing a considerable deficit in palliative care. Our study revealed a knowledge increase during medical residency, with superior performance among residents with ≥3 years of training, especially in the "opioid side effects" domain.

Moreover, our study demonstrated that few medical residents had the opportunity to participate in formal palliative care training at their medical schools or residence. Castro et al. revealed that palliative care training in Brazil is insufficient and is heterogeneous across regions.^(26)^ Nonetheless, our study showed that previous experience with palliative care was associated with higher PEACE-Q-Br scores. Participants who reported more extensive contact with patients in palliative care (>10 patients) over the previous 6 months performed better than those with less contact. Therefore, we hypothesized that increased exposure to opioids and palliative care within the hospital may lead to implicit learning and can explain this superior performance. A study conducted among medical residency applicants in Brazil showed that greater previous experience with dying patients was associated with higher overall palliative care knowledge.^(27)^This association is supported by a systematic review suggesting that interventions resulting in more extensive practical experience in palliative care effectively improve symptom management, including pain.^(28)^

Our study had some limitations. First, the instrument was validated at a single institution. Although the study site has a high diversity of medical residents, further studies are needed to assess the psychometric parameters of the PEACE-Q Br in different contexts. Second, the high exclusion rate due to nonresponse to the retest may have underestimated the reliability of the estimates. However, because the ICC was satisfactory, this limitation did not appear relevant to the validation process. Additionally, the low response rate could have introduced bias into the analysis of the residents’ performance. Notably, the focus of this study was to translate and validate a Brazilian version of the instrument, and the analysis of the medical residents’ performance was considered preliminary; hence, further exploration is required in future studies.

## CONCLUSION

In this study, we translated and adapted the PEACE-Q into Brazilian Portuguese, which resulted in acceptable internal consistency and moderate reliability. The translated version of the instrument (PEACE-Q Br) appears promising for evaluating palliative care knowledge among medical residents in Brazil and may help guide improvements in medical postgraduate studies.

## SUPPLEMENTARY MATERIAL

### Translation, cross-cultural adaptation, and validation of the Palliative Care Knowledge Questionnaire for PEACE (PEACE-Q) in Brazilian Portuguese

João Paulo Aureliano Silva, Aleida Nazareth Soares, Adriana Silvina Pagano, Cristiana Guimarães Paes Savoi, Alexandre Ernesto Silva, Alexandre Sampaio Moura

**DOI: 10.31744/einstein_journal/2025AO1243**

**Questionário versão final em português do Brasil do PEACE-Q**

Para cada afirmativa, escolha apenas uma resposta: primeira coluna V (aquela que considerar uma afirmação verdadeira) ou segunda coluna F (aquela que considerar uma afirmação falsa).

**Table:**

Afirmativas		V	F
Domínio: Filosofia dos cuidados paliativos			
	1. Cuidados paliativos é sinônimo de cuidados de fim de vida		
	2. Entre brasileiros, os principais sentimentos associados ao câncer são o medo da morte e da dor		
	3. O consumo de opioides para dor no Brasil é menor do que no México, na Argentina e no Chile		
Domínio: Dor oncológica			
	4. Quando a dor oncológica é intensa, um dos medicamentos do terceiro degrau da escada analgésica da OMS é usado como analgésico inicial		
	5. Quando se começa a prescrever opioides, todos os analgésicos não opioides devem ser descontinuados		
	6. A morfina é usada com segurança em pacientes com insuficiência renal		
	7. A dose de resgate de opioide é de 5% da dose diária total		
	8. Como não ocorre tolerância para náusea induzida por opioide, um antiemético deve ser prescrito a todos os pacientes		
	9. A dose diária total de opioides é aumentada em 10% se a dor não for aliviada		
	10. Deve-se considerar a rotação ou substituição do opioide, quando houver dificuldade para aumentar a sua dose, devido a efeitos adversos		
	11. Cerca de 10% dos pacientes com dor basal controlada têm dor do tipo breakthrough (escapes de dor)		
	12. Procedimentos dentários invasivos devem ser evitados durante o tratamento com bisfosfonatos		
Domínio: Efeitos colaterais dos opioides			
	13. Náusea e/ou vômito induzido por opioides ocorrem em 80% ou mais dos pacientes que fazem o uso destes		
	14. É necessário associar um laxativo aos opioides orais, porque a maioria dos pacientes que os utilizam apresenta constipação		
	15. Os opioides causam dependência em 0.2% ou menos dos pacientes com câncer sob monitoramento cuidadoso		
Domínio: Dispneia			
	16. Se um paciente tem dispneia, sua PaO_2_ é inferior a 60mmHg		
	17. A morfina é efetiva para a dispneia		
	18. Se a temperatura ambiente é mantida mais alta (quente), um paciente com dispneia frequentemente sente alívio		
Domínio: Náuseas e vômitos			
	19. Os neurotransmissores no centro do vômito são dopamina, histamina, acetilcolina e serotonina		
	20. Quando a principal causa da náusea é hipercalcemia, a administração de bisfosfonato é um tratamento útil para o alívio deste sintoma		
	21. A metoclopramida pode causar acatisia		
Domínio: Sofrimento psicológico			
	22. Quando um paciente tem um elevado nível de sofrimento psicológico, recomenda-se que os médicos avaliem se o paciente tem ideação suicida		
	23. Quando o paciente tem ideação suicida, recomenda-se avaliação psiquiátrica		
	24. Ansiolítico é um dos medicamentos úteis para pacientes em sofrimento psicológico		
Domínio: Delirium			
	25. O delirium ocorre devido a medicamentos ou causas orgânicas		
	26. Os benzodiazepinicos devem ser a primeira escolha para tratar o delirium		
	27. É melhor manter o quarto de um (a) paciente com delirium bem escuro, para que ele (a) possa dormir bem		
Domínio: Comunicação			
	28. Uma pergunta aberta é aquela que não pode ser respondida com um simples "sim" ou "não" e requer uma resposta livre baseada nos próprios sentimentos da pessoa		
	29. Quando os médicos comunicam más notícias, deveriam indagar quais são as preocupações e a compreensão do paciente sobre a doença		
	30. É melhor usar a palavra ‘câncer’ repetidas vezes ao informar ao paciente sobre a sua doença maligna		
Domínio: Comunicação			
	31. De acordo com o Ministério da Saúde, os cuidados paliativos deverão ser oferecidos em qualquer ponto da Rede de Atenção à Saúde do SUS, incluindo atenção primária		
	32. Os opioides são fornecidos pelo SUS aos pacientes em cuidados paliativos que necessitam dessa classe de medicamentos		
	33. Segundo o Ministério da Saúde, o paciente em cuidado paliativo, portador de câncer, deve ter assistência médica de urgência/emergência disponível 24 horas por dia na instituição que esteja matriculado		
